# Synchronous functional magnetic resonance eye imaging, video ophthalmoscopy, and eye surface imaging reveal the human brain and eye pulsation mechanisms

**DOI:** 10.1038/s41598-023-51069-1

**Published:** 2024-01-26

**Authors:** Seyed-Mohsen Ebrahimi, Johanna Tuunanen, Ville Saarela, Marja Honkamo, Niko Huotari, Lauri Raitamaa, Vesa Korhonen, Heta Helakari, Matti Järvelä, Mika Kaakinen, Lauri Eklund, Vesa Kiviniemi

**Affiliations:** 1 Oulu Functional NeuroImaging (OFNI), Diagnostic Imaging, Medical Research Center (MRC), Finland Oulu University Hospital, 90029 Oulu, Finland; 2 Research Unit of Health Sciences and Technology (HST), Faculty of Medicine, University of Oulu, 90220 Oulu, Finland; 3 Department of Ophthalmology and Medical Research Center, Oulu University Hospital and Research Unit of Clinical Medicine, University of Oulu, Oulu, Finland; 4 Oulu Center for Cell-Matrix Research, Faculty of Biochemistry and Molecular Medicine, Biocenter Oulu, University of Oulu, Oulu, Finland

**Keywords:** Neuroscience, Diseases, Medical research, Optics and photonics, Physics, Optical physics

## Abstract

The eye possesses a paravascular solute transport pathway that is driven by physiological pulsations, resembling the brain glymphatic pathway. We developed synchronous multimodal imaging tools aimed at measuring the driving pulsations of the human eye, using an eye-tracking functional eye camera (FEC) compatible with magnetic resonance imaging (MRI) for measuring eye surface pulsations. Special optics enabled integration of the FEC with MRI-compatible video ophthalmoscopy (MRcVO) for simultaneous retinal imaging along with functional eye MRI imaging (fMREye) of the BOLD (blood oxygen level dependent) contrast. Upon optimizing the fMREye parameters, we measured the power of the physiological (vasomotor, respiratory, and cardiac) eye and brain pulsations by fast Fourier transform (FFT) power analysis. The human eye pulsated in all three physiological pulse bands, most prominently in the respiratory band. The FFT power means of physiological pulsation for two adjacent slices was significantly higher than in one-slice scans (RESP1 vs. RESP2; df = 5, p = 0.045). FEC and MRcVO confirmed the respiratory pulsations at the eye surface and retina. We conclude that in addition to the known cardiovascular pulsation, the human eye also has respiratory and vasomotor pulsation mechanisms, which are now amenable to study using non-invasive multimodal imaging of eye fluidics.

## Introduction

Despite the high metabolic activity of retinal and brain tissues, and their consequent requirement for fluid homeostasis and metabolite clearance, lymphatic vessels are absent from the eye and brain. However, recent work shows that the eye, much like the brain, is endowed with a glymphatic drainage system that mediates metabolite and fluid clearance along perivascular spaces adjacent to the optic nerve (ON)^1^. The glymphatic brain solute transport system convects solutes along perivascular cerebrospinal fluid (CSF) spaces, being driven by physiological pulsations that predominate during sleep^2,3^. Indeed, the glymphatic system of the murine eye can remove injected tracers, as driven by intracranial pressure differences between the eye and the intracranial space^4^. In human patients with normal pressure hydrocephalus (NPH), opposing movements of magnetic resonance imaging (MRI) tracer have been detected around the ON after intrathecal administration of Gd^3+^ contrast medium^5^. However, intra-ocular tracer injection studies are not clinically feasible for the human eye, and, in any event, the tracer molecules would likely impair glymphatic convection by obstructing microscopic structures in the ON, especially in patients with disorders affecting eye solute transfer and pressure^6^. Furthermore, MRI tracer studies are unfit to detect the physiological drivers affecting glymphatic convection in the human eye or brain. This technical limitation calls for developing faster, non-invasive, and tracer-free methods to image and monitor glymphatic systems^7^.

Functional MRI (fMRI) utilizing the T2* weighted signal offers just such a non-invasive, tracer-free approach to investigate the driving forces of CSF solute transport in the human brain. Ultrafast MR encephalography (MREG) has recently indicated that CSF convection in human brain is affected by three drivers, namely very low frequency (VLF), respiratory (RESP), and cardiac (CARD)^8^ pulsatility, each of which increases in power during slow wave sleep in brain regions of increased CSF convection^9^. Importantly, the physiological pulsations are differentially involved in diverse neuropathologies, including Alzheimer’s disease^10^, epilepsy^11^, primary central nervous system (CNS) lymphoma^12^ and narcolepsy^13^. However, the ultrafast MREG spiral acquisition scans established for brain are not optimized for eye tissue characterization.

Echo planar imaging (EPI) of blood oxygen level dependent (BOLD) contrast fMRI has been used to correlate brain function to eye gaze, pupil dilation, and eyelid closure as markers of numerous brain functions, including attention, sympathetic activity, and vigilance^14^. Applications of the functional BOLD fMRI of eye tissue (fMREye) technique have hitherto focused on effects of voluntary eye movement and eyelid opening and closure^15^. However, Duong and colleagues have pioneered a retinal BOLD imaging method that reveals classic hyperemic BOLD responses in the retina during visual stimuli and physiological challenges^16^. EPI in single or adjacent slices can be imaged with high in-plane resolution to enable the delineation of small intraocular structures, also with sufficiently high temporal resolution (100 ms) to sample physiological pulsations without need for aliasing correction^17^. Despite its non-invasive character and the wide availability of fMRI, there are no reports regarding the physiology of human eye pulsatility as revealed with BOLD contrast imaging.

We present novel findings on physiological pulsations involved in solute transport in the human eye, complementing previous studies in the field of ophthalmology that have utilized concurrent methodologies^18–26^. Previous studies employing interferometry (e.g., Laser doppler holography. optical coherence tomography, scanning laser ophthalmoscopy), video-ophthalmoscopy, and subsequent processing methods have demonstrated cardiac cycle-induced pulsations in various eye structures, including the cornea, retina, optic disc, and retinal vessels, and highlighted the influence of factors such as heart rate, aging, intraocular pressure, and intracranial pressure on these eye structures pulsations^18,21–28^. Additionally, respiratory frequency pulsations have been observed in retinal video recordings^19^. We developed and optimized a fast fMREye scanning sequence with a repetition time of 100 ms to detect pulsations in the eye and retrobulbar segment of the optic nerve (ON). To validate and quantify these physiological eye pulsations, we employed two MRI-compatible procedures in addition to conventional measures of cardiorespiratory physiology (respiratory belt to verify respiratory pulsation and cardiovascular SpO2 measurement for cardiac pulsation verification.).

First, we utilized a functional eye camera (FEC), as commonly used for eye/pupil tracking in fMRI studies, to detect pulsatility in the corneal surface, synchronized with the fMREye measurements^29^. Eye tracking by EEG^30^ or an MR-compatible video camera^31^ are commonly employed to monitor pupil diameter, gaze direction, or vigilance during MRI^32^. MR-compatible eye tracking cameras record gaze position during scanning with high temporal and spatial resolution, and hence enable the investigation of gaze-related brain activity^33^. However, we consider this technology to augment importantly the scope of fMRI investigations. Second, we further augmented the FEC with advanced optics to provide a dedicated MRI-compatible video ophthalmoscope (MRcVO), which we used to scan retinal pulsations during fMREye imaging^34^. Our optimization of fMREye imaging parameters minimized interference with MRcVO and FEC camera signals. Using this multimodal imaging approach, we confirmed the presence of all three physiological pulsation frequency bands, with reference to the conventional physiological monitoring data.

## Results

### Number of slices

**Figure 1:**
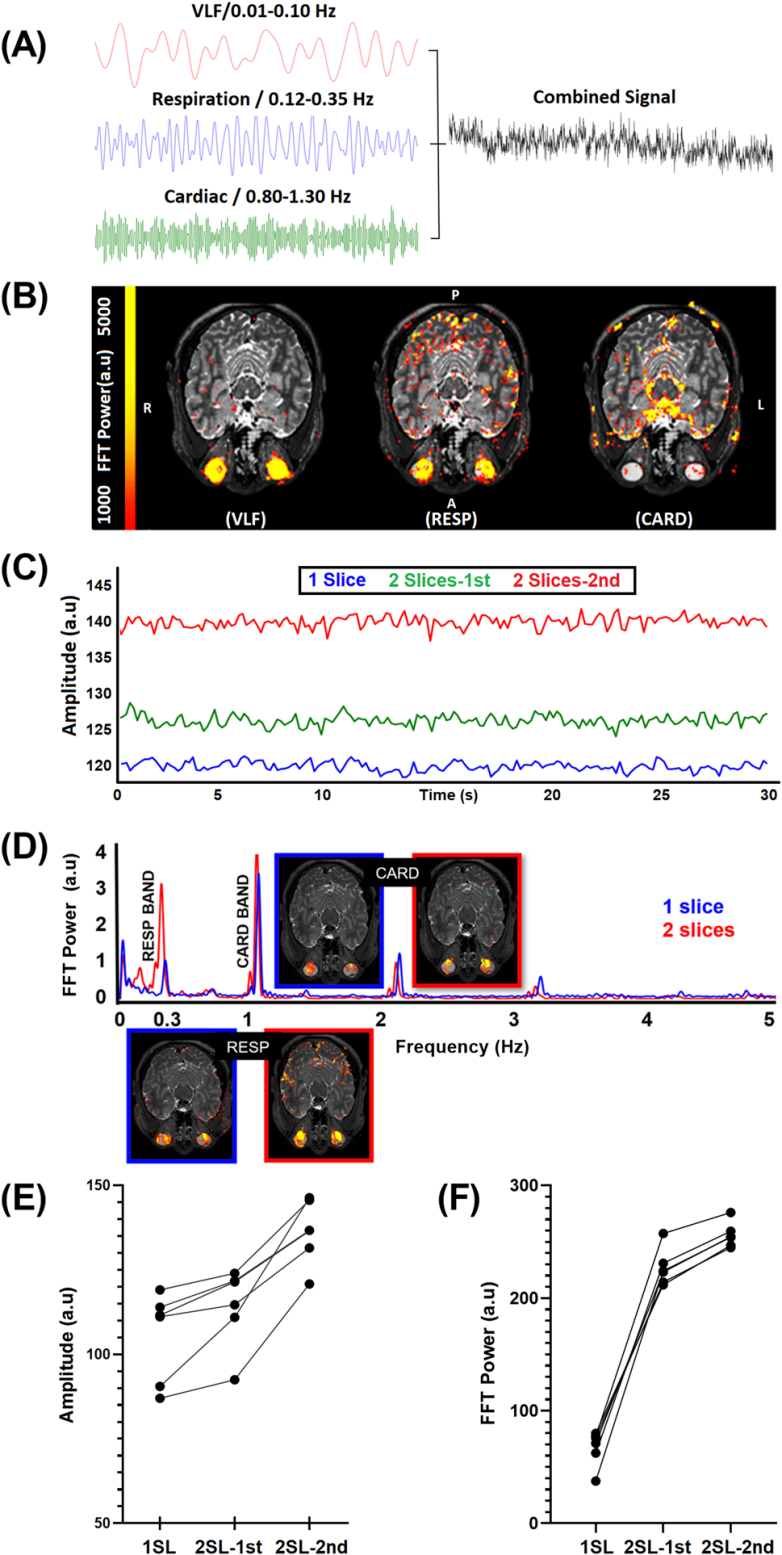
(**A**) A single-subject example of raw fMREye signal with filtered very low frequency (VLF 0.01–0.1 Hz) and individual bands for respiration (RESP 0.12–0.35 Hz) and cardiovascular pulsations (CARD 0.8–1.3 Hz), which were 0.1 Hz wide and centered around the individual peaks (i.e., peak ± 0.05 Hz). (**B**) An example of the individual FFT power map of the three physiological pulsations VLF, RESP, CARD). (**C**) The mean fMREye time signal example from a whole image of head slices with a single slice data (1SL) and adjacent slices from two-slice (2SL) data, where the two-slice data signal was of higher baseline intensity level than the one slice scan. (**D**) The mean FFT power plot for the whole head fMREye image taken in the same representative subject, showing power peaks in three physiological bands from 1 and 2SL data. (**E**) the mean signal intensity level of 1SL, 2SL-1st, 2SL-2nd slice data of whole head indicates that in each case the signal intensity is lower in 1SL than in 2SL data, and also that the second slice always had a higher magnitude. (**F**) The mean FFT power of the 0 to 5 HZ band of a whole head data indicates that the power likewise increased from 1 to 2SL data, and that 2nd slice has highest powers.

First, we set about to optimize the number of eye slices, aiming to minimize signal artifacts and to maximize signal power. The original signal is a combination of VLF, RESP, and CARD signals (Fig. 1A). Next, we investigated whether fMREye could also detect all three physiological pulsation types from intracranial space. From the whole image of head slice fMREye data, the FFT power spectra could indeed synchronously identify the three distinct pulsation bands (Fig. 1B,D), namely the VLF, respiratory and cardiovascular frequencies as known from earlier studies^8,9^. To optimize the number of slices within the ultrafast 100 ms TR (Repetition Time) scanning timeframe, we obtained recordings in single and adjacent pairs of slices. Subjects were scanned after pupillary dilation of the left eye in MRcVO (n = 12) and without dilation in FEC (n = 6). The two slice datasets had significantly higher mean BOLD signal intensity level and pulsation power (Fig. 1D–F). For each of the three pulsations bands, the peak of FFT power for a pair of adjacent slices exceeded that of single slice recordings. Thus, the fMREye scan robustly detected all three physiological head (both intra and extracranial) pulsations, i.e., vasomotor, respiratory, and cardiovascular pulsations, c.f. Figure 1E.

Figure 1C shows the fMREye time signal averaged from one subject, occurring repeatedly across all six subjects. Our analysis of the mean signal intensity levels showed that the 1 slice data signal amplitude was lower than the 2-slice data in each of the six subjects, c.f. Fig. 1E. We also calculated the mean FFT power for one slice versus two slice whole head data, finding that VLF was 68.11 ± 28.73 (VLF_1_) versus 103.1 ± 28.24 (degrees of freedom (df) = 5, p = 0.026) for VLF_2_.; Corresponding values for RESP were 90.23 ± 27.14 versus 212.4 ± 90.48 (df = 5, p = 0.045), and corresponding values for CARD were 143.6 ± 34.70 versus 241.4 ± 42.57 (df = 5, p = 0.002). All p values are reported with Bonferroni correction for multiple comparisons. One might suspect that the number of slices does not affect the mean signal or the FFT power results in fMRI data. However, quite surprisingly to us, after measuring it individually, two slice scan data always had greater mean signal intensity level of the time signal and more power to FFT and more in depth the 2nd slice had highest signal level and power also (Fig. 1E,F).

### MR-compatible MRcVO and FEC camera signal interference

**Figure 2:**
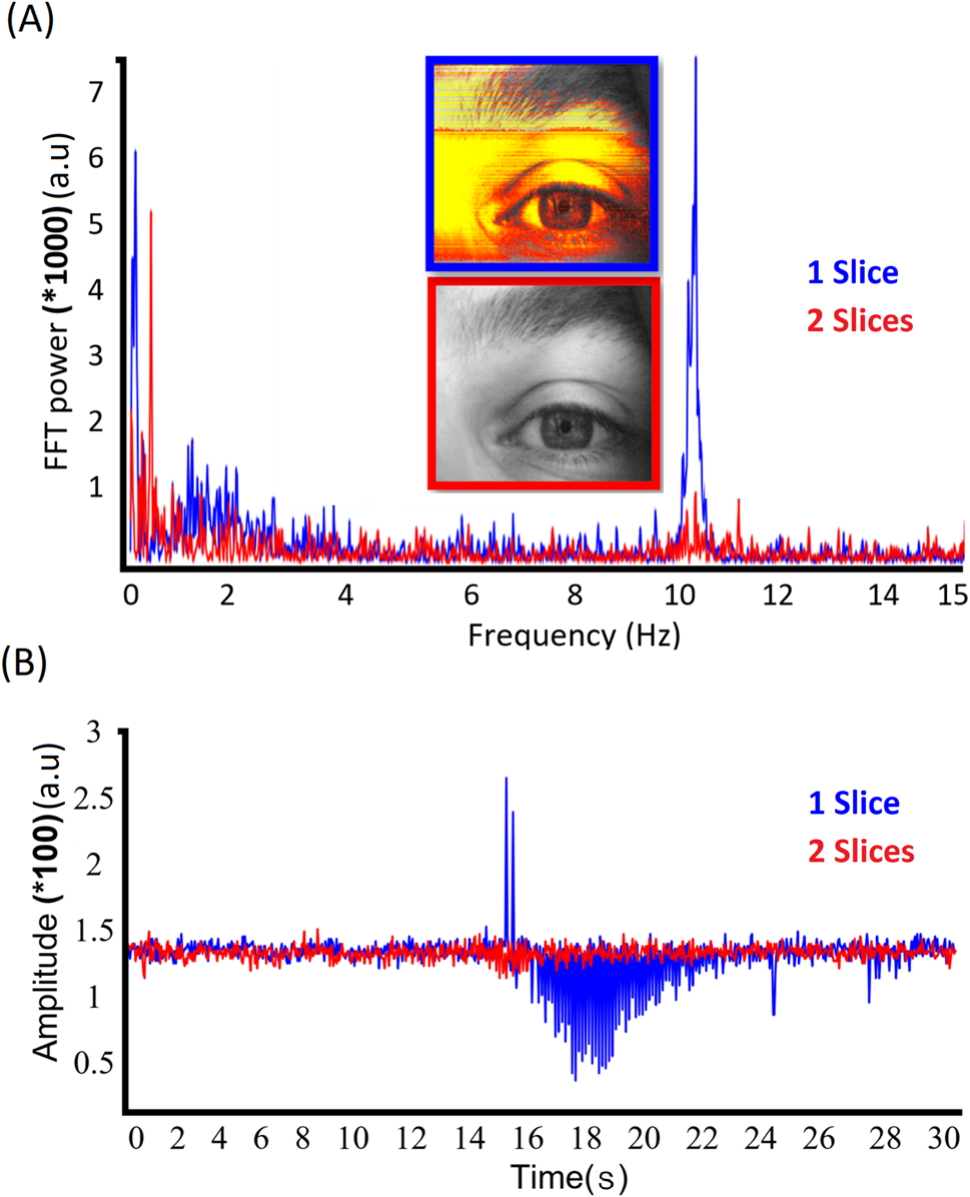
Lower image flicker artifact in two slice mode on eye images obtained by FEC during the fMREye scan (chart illustration of one subject). (**A**) FFT power chart shows in the 10 Hz frequency band a high noise power in the one slice scan FFT spectrum, which is almost absent in the corresponding two slice FFT spectrum. (**B**) A 30 s time signal for the eye corneal surface FEC video data, illustrating artifactual flicker for the single slice (blue) (fMREye signal compared to the more stable two slice fMREye scan (red).

We next investigated the effect of different slice numbers on the MR-compatible eye camera signals. Single slice fMREye scanning had a large artifact effect in the FEC/MRcVO camera signal compared to two slices fMREye data, as shown in Fig. 2.

The one slice signal data had markedly greater flicker in the video recording (Fig. 2B), with a peak amplitude for the flicker artifact at 10 Hz in the FFT power spectrum. However, this artefact was clearly absent in the 2-slice data, which we therefore selected for further analysis.

### Slice thickness optimization

To optimize the slice thickness, we scanned the fMREye data with slice thickness 3, 4 and 5 mm. Obviously, thinner slices have better spatial resolution because of their smaller voxel size and lesser effects of partial volume. Also, MRI resolution for two slices of 3 mm slice thickness would certainly be better than for one slice of 6 mm slice thickness, but with comparable coverage of the ON.

**Figure 3:**
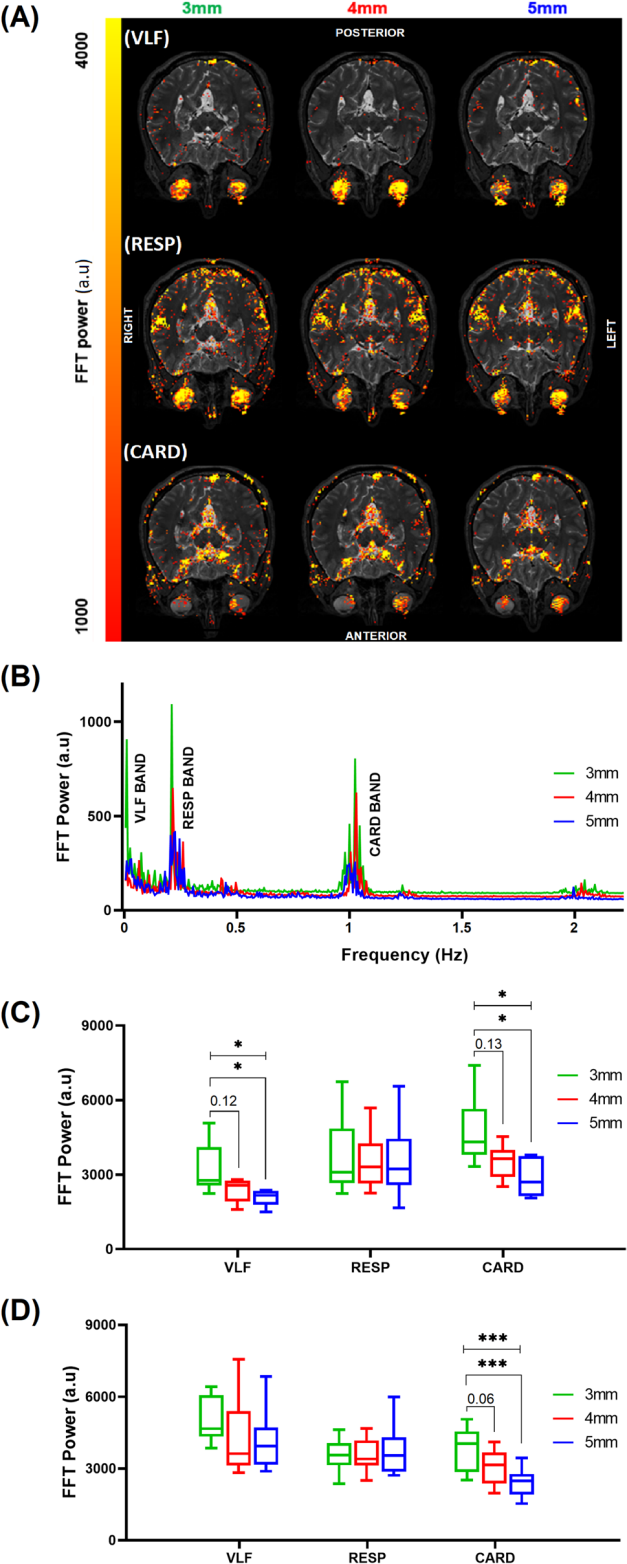
fMREye scan detects vasomotor (0.01–0.1 Hz), respiratory (0.25 ± 0.05 Hz), and cardiovascular (1.1 ± 0.05 Hz) pulsations from whole head slices image data. (**A**) A representative FFT power map of the physiological pulsation from two slice data of 3-, 4-, and 5-mm slice thicknesses. (**B**) The mean FFT power of the same subject’s whole fMREye image for all scan protocols illustrate clear VLF, RESP, and CARD peaks and cardiorespiratory harmonics (N = 1). (**C**) The FFT power spectrum in different slice thickness from whole head slices fMREye simultaneous FEC, without pupillary dilation. The mean FFT power in the cardiac band was significantly higher for thinner slices, and likewise in the VLF band for 3 mm slices compared to 5 mm. There were no significant differences in the RESP band (N = 12). (**D**) The FFT power spectra from head slices fMREye data of different slice thickness, obtained simultaneously with MRcVO after pupillary dilation. FFT power for cardiac pulsation was higher for thinner slice scans compared to 5 mm thickness (N = 12). (* < 0.05, ** < 0.01, *** < 0.001, **** < 0.0001).

As shown in Fig. 3, we investigated the effects of slice thickness on FFT power and signal amplitude. Thinner slices proved to have stronger signal power and FFT power amplitude. We found significant differences for CARD results in both conditions (whole eye and brain scanned simultaneously by fMREye, along with eye scanning by FEC and retina scanning by MRcVO), and VLF results for fMREye simultaneous FEC, but no significant results for RESP according to the ANOVA statistical test results. Inspection of data for the whole image shows conspicuously higher power in the VLF and cardiac bands for 3 mm slice thickness compared to 4- or 5-mm thickness. In this population, we saw no effect of slice thickness on the respiratory frequency-related pulsation FFT power.

Mean (SD) FFT power values (n = 6 subjects, df = 5) in whole image FEC and fMREye data for VLF were 3128 ± 1043 at 3 mm (VLF_3_), 2299 ± 479.0 at 4 mm (VLF_4_), and 1986 ± 330.1 at 5 mm slice thickness (VLF5_5_) (ANOVA test, p = 0.032). VLF_3_ vs. VLF4 (Tukey test, p = 0.12), VLF_3_ vs. VLF_5_ (Tukey test, p = 0.03). Corresponding mean (SD) FFT powers were 3998 ± 1627 for RESP_3_, 3814 ± 1217 = for RESP_4_, and 3842 ± 1646 for RESP_5_ (ANOVA test, p = 0.97). Corresponding mean (SD) FFT powers were 49,229 ± 1435 for CARD_3_, 3723 ± 687.9 for CARD_4_, and 3051 ± 750.0 for CARD_5_ = (ANOVA test, p = 0.018). CARD_3_ vs. CARD_4_ (Tukey test, p = 0.133), CARD_3_ vs. CARD_5_ (Tukey test, p = 0.015), Fig. 3C.

In the whole image MRcVO and fMREye data, the mean (SD) FFT powers (n = 12 subjects, df = 11) were 5079 ± 878.6 for VLF_3_, 4303 ± 1500 for VLF_4_, and 4161 ± 1206 for VLF_5_ (ANOVA test, p = 0.18). VLF_3_ vs. VLF_4_ (Tukey test, p = 0.31), VLF_3_ vs. VLF_5_ (Tukey test, p = 0.20). Corresponding mean (SD) of FFT powers were 3509 ± 666.2 for RESP_3_, 3538 ± 660.9 for RESP_4_ and 3833 ± 1095 for RESP_5_ (ANOVA test, p = 0.61). Corresponding mean (SD) of FFT powers were 3769 ± 865.6 for CARD_3_, 3070 ± 728.2 for CARD_4_, and 2410 ± 558.4 for CARD_5_ = (ANOVA test, p = 0.0003). CARD_3_ vs. CARD_4_ (Tukey test, p = 0.062), CARD_3_ vs. CARD_5_ (Tukey test, p = 0.0002), Fig. 3D.

### Optic nerve (ON)

**Figure 4:**
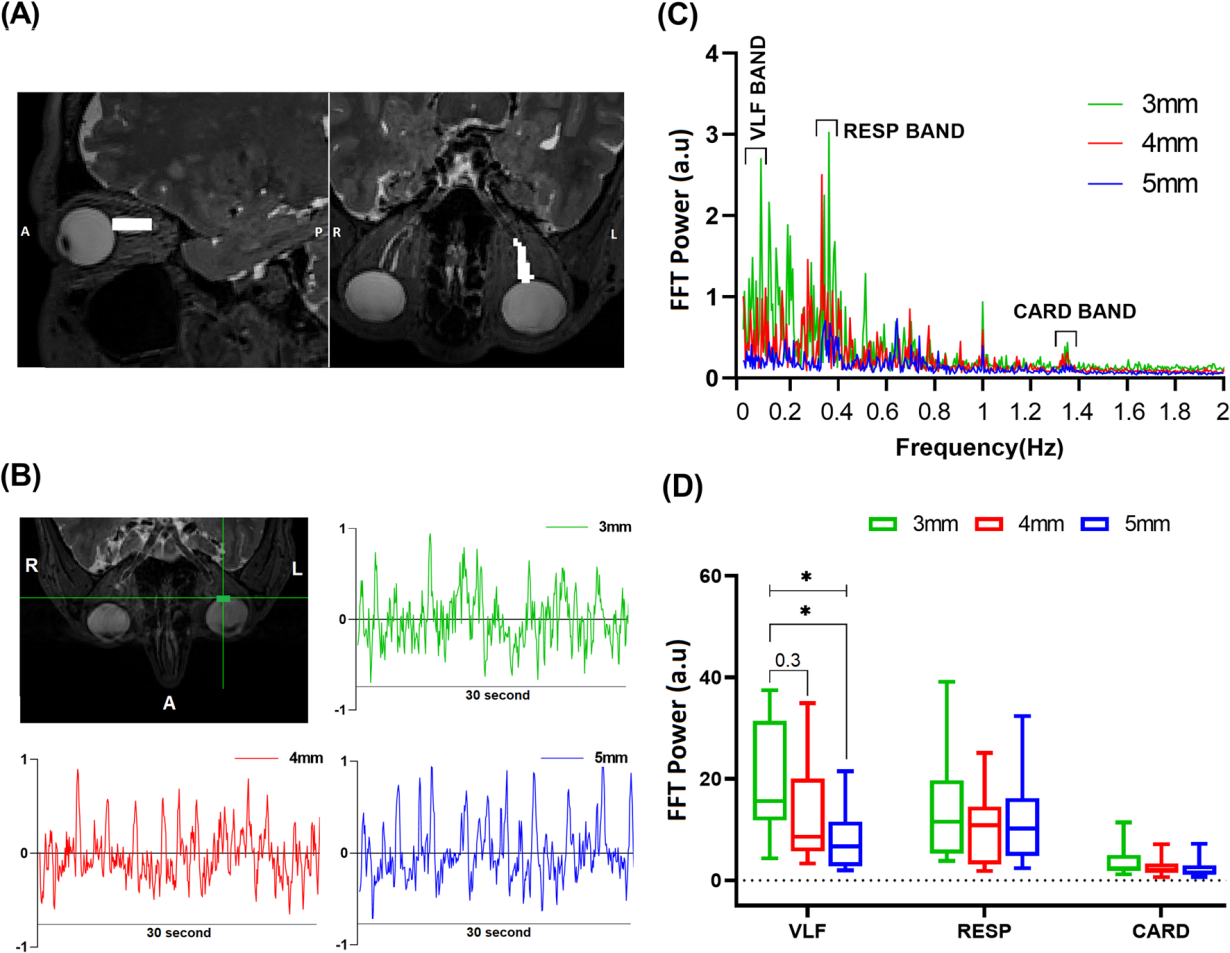
(**A**) The ROI map highlighting the optic nerve (ON). The navigation abbreviations are posterior (P), anterior (A), left (L), and right (R); (**B**) The fMREye time signal from a single voxel in the retina, comparing results with different slice thicknesses; (**C**) The FFT power mean chart of the ON, demonstrating a stronger and clearer power peak in the VLF and RESP band for a 3 mm slice thickness in comparison to 4 mm and 5 mm (N = 1); (**D**) Group results of the FFT power for ON pulsations, indicating significantly higher power in the VLF band for a 3 mm slice thickness in contrast to 4 mm and 5 mm (N = 12) (test p-value: *p < 0.05).

The paravascular spaces within the optic nerve are important channels for glymphatic eye clearance^35^. Therefore, we analyzed specifically the ON in fMREye scan data. Figure 4 depicts a representative ON region of interest (ROI) projected on the structural MR image.

The slices thicker that 3 mm usually extended beyond the ON ROI, thus being vulnerable to partial volume effects from surrounding tissue. The three pulsation bands from the fMREye data indicated significantly higher power in the VLF and CARD bands for slices of 3 mm thickness. The respiratory pulsations tended to have the lesser dependence in slice thickness (as also seen for the retina, above), but without reaching significance. Ten among the twelve subjects (due to lacking ON coverage for two subjects) served for investigation of the ON (retrobulbar segment of ON) pulsations. One-way ANOVA showed no significant effect of slice thickness for RESP results. Mean (SD) FFT power (N = 10 subjects, df = 9) were 19.67 ± 11.36 for VLF_3_, 12.87 ± 10.34 for VLF_4_, and 7.73 ± 6.24 for VLF_5_ (ANOVA test, p = 0.046), with VLF_3_ vs. VLF_4_ (Tukey test, p = 0.3) and VLF_3_ vs. VLF_5_ (Tukey test, p = 0.036). Mean (SD) of FFT power was 14.44 ± 10.85 for RESP_3_, 10.45 ± 7.47 for RESP_4,_ and 11.81 ± 9.24 for RESP_5_ (ANOVA test, p = 0.62). Mean (SD) of FFT power was 3.74 ± 3.14 for CARD_3_, 2.73 ± 2.02 for CARD_4_, and 2.35 ± 2.03 for CARD_5_ (ANOVA test, p = 0.43). CARD_3_ vs. CARD_4_ (Tukey test, p = 0.63), CARD_3_ vs. CARD_5_ (Tukey test, p = 0.42), Fig. 4D.

### Human video ophthalmoscopy and fMREye BOLD signal

**Figure 5:**
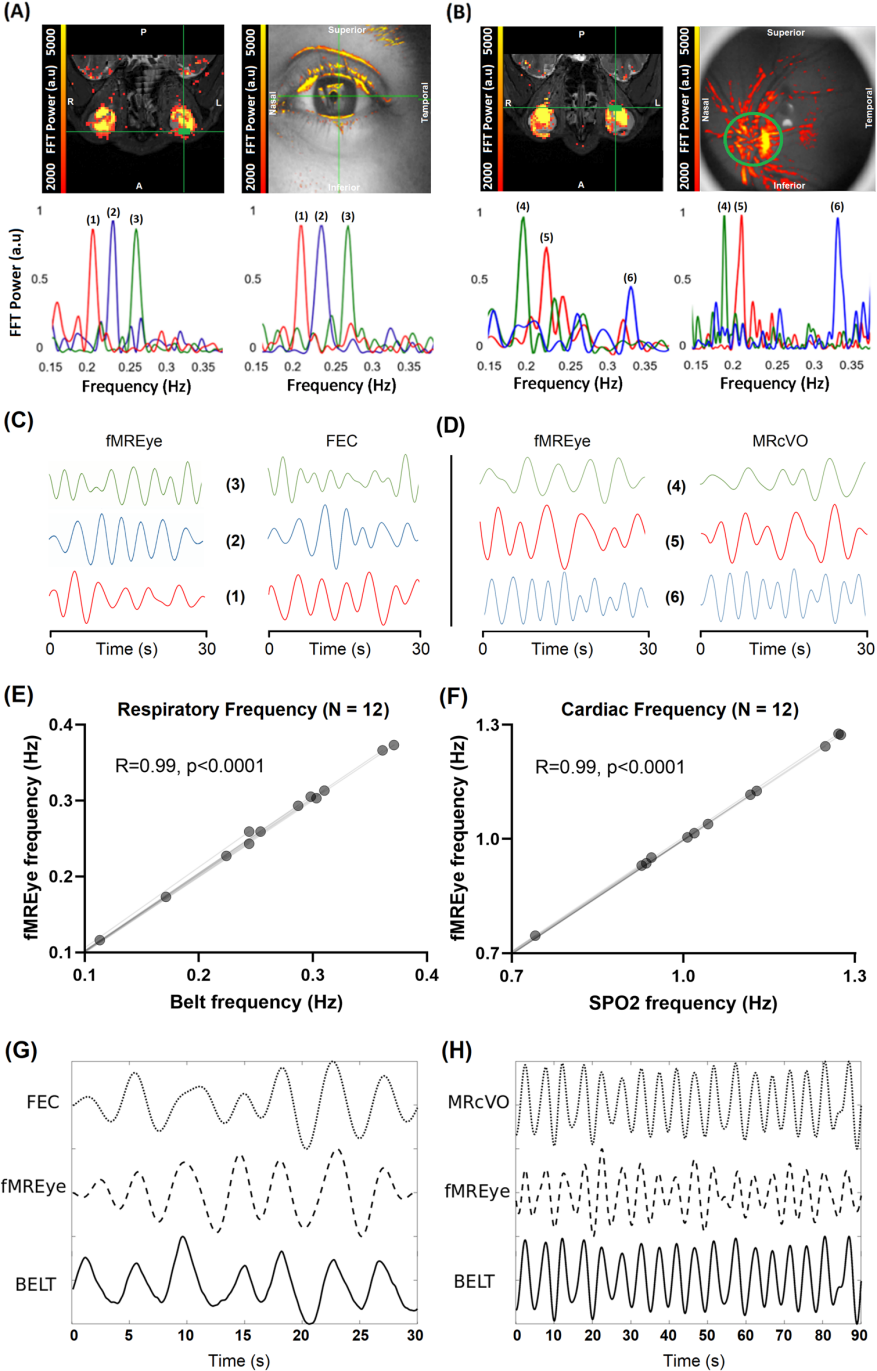
(**A**) Simultaneous fMREye and FEC surface video scanning reveals strong respiratory eye pulsations at identical frequencies in each of three subjects. The whole (left) eye and pupil pulsates in the respiratory frequency. The navigation abbreviations are posterior (P), anterior (A), left (L), and right (R); (**B**) Simultaneous fMREye and MRcVO also detected strong respiratory range pulsations in the human eye. Notably, both the whole (left) eye structure as well as the retinal vasculature pulsated strongly at identical respiratory frequency ranges in all three subjects. (**C**) Time-courses for comparing fMREye and FEC signal in for 3 subjects in RESP frequency range. (**D**) RESP signal time-courses of fMREye and MRcVO data for comparison in 3 subjects. (**E**) Respiratory belt data and (**F**) cardiovascular SpO_2_ showed identical physiological frequency peaks compared to the fMREye whole image FFT spectra. (**G**) Example correlation coefficient between the respiratory frequency fMREye ON signal and MR respiratory monitoring belt was 0.79, and the correlation coefficient between the FEC iris respiratory frequency signal and MR respiratory monitoring belt was 0.78 measured from one subject. (**H**) Example correlation coefficient between the respiratory frequency fMREye ON signal and MR respiratory monitoring belt was 0.89, and the correlation coefficient between the MRcVO (one pixel from the optic disk) respiratory frequency signal and MR respiratory monitoring belt was 0.68 measured from one subject. The eyelid blinks may reduce the correlation values in MRcVO.

To verify the existence of physiological eye pulsations, we interrogate the MRcVO and FEC scans. As expected, we indeed found synchronous eye and brain pulsations in the respiratory band for three individuals extending from the ocular surface to the back of the eye, as shown by fMREye and FEC, and by MRcVO imaging. The MRcVO and FEC results both confirmed that the eye does indeed pulsate synchronously with the fMREye pulsations within the respiratory band, c.f. Fig. 5. Only 3 of 12 cases were successful for MRcVO, whereas 3 of 6 cases were successful for FEC in detecting the respiratory pulse. Some datasets were excluded due to eye and head motion, and some were unusable due to other artifacts and camera interference flickers.

Due to head motion and eye saccades, the MRcVO data were generally unsuitable for physiological FFT power analysis. *Mcflirt* motion correction of nifti-files of MRcVO data failed to align the dynamic saccade results to a satisfactory extent (c.f. supplementary MRcVO video). Therefore, the video data were cropped as a circle around the optic disk and a 1 to 2 mm radius that included retinal surface and vessels. Furthermore, for motion correction and registration of the retinal images, we employed an open-source GitHub code specifically designed for retinal image registration (https://github.com/umn-milab/retinaimagingtoolbox) for more reliable analysis. FEC data suffered less from eye saccades and therefore sufficed to capture pulsations. However, the CARD pulsation bands were not detectable in fMREye.

Figure 5A,B presents the results of three cases in comparing eye surface imaging with fMREye scanning, and their use for comparing the RESP band of eye pulsation in the back of the eye by MRcVO and fMREye. After comparing the fMREye results with respiration belt and oximeter recordings, the FEC and MRcVO data were also compared to the fMREye imaging data, revealing consistent peaks in the RESP band. Simultaneous fMREye and MRcVO examinations further detected robust pulsations within the respiratory range in the human eye. This pulsatility was observed both in the eye surface and the retinal vasculature, with matching peak frequencies in the fMREye and FEC/MRcVO recordings (The supplementary, Table 2). To understand better the respiratory signal, we filtered this band, which revealed the time-courses of inter-modality for fMREye and FEC datasets and for fMREye and MRcVO datasets for 3 subjects in 3 mm thickness slice (Fig. 5C,D). The respiratory and cardiac pulsations results achieved from fMREye were confirmed by respiratory belt data and cardiovascular SpO2 for twelve subjects (Fig. 5E and F), illustrating identical physiological frequency peaks compared to fMREye whole image FFT spectra. Statistical analysis for both graphs showed p < 0.0001 and Spearman’s correlation (R) equal to 0.99. The time domain correlation coefficients between the respiratory frequency signal obtained from the fMREye ON and the respiratory belt were 0.79 and between FEC iris and respiratory belt 0.89, respectively form an example subject (Fig. 5G). Similarly, the correlation coefficients between the respiratory frequency signal derived from the fMREye ON and the respiratory belt was 0.78 and between MRcVO (a single pixel from the optic disk) and the respiratory belt was 0.68 (Fig. 5H). It's worth noting that the presence of eyelid blinks may have a diminishing effect on the correlation values in the MRcVO signal. The temporal alignment of the signals shown in Fig. 5G is illustrative only and may not truly reflect real-time synchronization due to the lack of a millisecond-accurate synchronization trigger (Fig. 5G,H).

## Discussion

In this study, we investigated new hybrid methods for measuring physiological pulsations of the eye using functional eye MRI BOLD imaging (fMREye), simultaneously with a functional eye camera (FEC) and MR-compatible video ophthalmoscope (MRcVO). Our optimization of the scanning procedures showed that 2-slice fMREye imaging with thin (3 mm) slices presented the least artifacts and was most sensitive in detecting pulsation powers in the eye. We further verified that the fMREye pulsation data precisely reflected physiological cardiorespiratory recordings from both the eye and brain, as further substantiated by peripheral measurements. Compared to the brain pulsation data, the ON had strong VLF and respiratory pulsations, but surprisingly small cardiovascular pulsation, even though the cardiovascular pulsations were clearly detectable simultaneously in the brain tissue, CSF, and also in the skin blood vessels. This result was also suggested by FEC and MRcVO data, wherein neither the human eye surface nor retina presented strong significant cardiac pulsation.

The successful imaging of physiological drivers of glymphatic solute transport in the eye called for specific technical improvements due to the special characteristics of the eye with respect to MRI. Advances in MRI technology and image processing such as image signal despiking enable imaging of the human eye with high spatiotemporal resolution^36^. In this study we optimized 3T conventional gradient recalled echo planar imaging parameters to quantify the power of physiological pulsations from the head structures, I.e., from the brain, CSF, skin and eye structures. To the best of our knowledge, this is the first study to systematically measure human eye pulsations with fMREye.

To our surprise, 2-slice data with thin slices exhibited the strongest FFT power results, making it the optimal choice for imaging pulsations (see Figs. 1 and 3). This unexpected outcome is likely attributable to both technical and physiological factors. Interslice crushing, shimming, and RF-transmitter inhomogeneities may have altered mean signal levels, while blood/CSF inflow effects likely increased significantly between 1-slice and 2-slice imaging. This issue is less relevant in multislice 3D data that covers the entire brain but becomes more pronounced when working with smaller volume slices typical of the eye an ON. Notably, the 2-slice ep2d approach provided the most sensitive imaging results for pulsations at 3 mm spatial resolution, most likely due to smallest partial volume effects in the 3 mm slices. In the future, using a thin multislice slab with < 1 mm cubic spatial resolution would likely yield still more accurate detection, especially of cardiovascular pulsations. Additionally, a 2–3 cm slab would facilitate alignment with MNI 305 space coordinates and motion correction. Since smaller slices and voxels are also less prone to partial volume effects, which mix tissue and fluid compartments, smaller voxel sizes per volume can yield better FFT power results.

We detected all three of the expected physiological pulsation frequencies in the human eye (Fig. 4). Previous fMRI studies of the eye aimed to assess the brain effects of what might be called general eye activity such as eyelid closure^37^ and saccadic movements, both of which proved to modify brain neuronal network connectivity^38^ and the amplitude of resting state fluctuations^39^. The eyelid posture reflects vigilance state, which has marked global effects on brain function, and is related to the emergence of widespread co-activation patterns after sleep onset^14^. Consensual changes in the eye’s pupil size are strongly associated with neuromodulator tone. More precisely, pupillary diameter is controlled by sympathetic and parasympathetic systems, and is recognized as a peripheral index of arousal in addition to its well-known regulation by ambient illumination^40^. The pupil dilation reflects the balance of effects arising from multiple brain regions, such as the Edinger-Westphal nucleus, hypothalamus, and locus coeruleus^41^.

Mostly likely due to limitations in temporal and spatial resolution, we were unable to detect all cardiac pulsation bands within the eye area, even though they can be readily seen visually in ophthalmological examinations and indeed form the basis of detecting papillary edema. However, we achieved robust recording of all physiological pulsation bands, including the cardiovascular pulsations both inside the brain and in extracranial structures such as temporal and facial skin arteries in all subjects. For this reason, we suspect that additional pulsations from eye saccade movements and other eye pulsation mechanisms may hinder the detection of the cardiovascular pulses in the eye with fMREye. This may be due to a) partially technical noise sources, b) simply still insufficient spatiotemporal resolution and c) the co-existence of other physiological impulses such as eye-saccades that mask the cardiac pulsations in the eye structures.

Nevertheless, fMREye offers unique advantages over established dynamic optical imaging methods like video-ophthalmoscopy, optical coherence tomography (OCT), or Doppler holography. While these optical methods provide higher temporal and, in some cases, higher 3D spatial resolution for the retinal structures^20^, fMREye can probe optic nerve and CSF pulsations inside and around the nerve, currently there may not be other methods that can do this. As shown by the Nedergaard group, the ON and it’s perineural CSF space is a key glymphatic pulsation area for waste removal^1^. Observations of the optic nerve head alone may not represent the convection and pulsation physiology of the whole optic nerve, nor its entry into the cranium. Furthermore, the ON glymphatic was already shown to have strong influence from intracranial pressure inducing CSF gradients of convection in the Wang et al., study^1^. Also, the driving effects of intracranial CSF pulsation on ocular structures cannot be readily measured using ophthalmic methods alone. fMREye offers several distinct advantages of detection simultaneously VLF waves and respiratory pulsations that are important in physiological^9,42^ and pathological processes^10–13,43^ within the CNS, as these three pulsations also module each other.

### Non-invasive and tracer-free

fMREye is a non-invasive imaging technique that does not require contrast agents. This eliminates the potential interference or alteration of the physiological processes being studied, ensuring a more natural and reliable measurement^12,44^.

### Complementary information

While established optical imaging methods excel with respect to spatial and temporal resolution, fMREye provides a unique perspective on the physiological pulsations driving solute transport. By integrating fMREye with other optical imaging modalities, researchers can gain a more comprehensive understanding of ocular physiology and potentially uncover novel relationships and mechanisms^2,3^.

### MRI technique

fMREye is an MRI technique, allowing simultaneous acquisition of functional and anatomical information. This integration enables researchers to correlate the physiological pulsations observed in fMREye with other brain and eye imaging data, facilitating a deeper understanding of the functional connectivity and interplay between these regions.

### Potential clinical applications

The development and optimization of fMREye holds promise for clinical applications. By elucidating the physiology of eye pulsatility, fMREye may aid in the diagnosis and monitoring of various ocular and neurological conditions, potentially providing valuable biomarkers for disease progression and treatment evaluation. Some of these diseases are glaucoma, Idiopathic Intracranial Hypertension (IIHT), neuromyelitis, and some optic nerve diseases^35,45,46^.

Several rhythmic or diurnal processes affect the eye. We note the diurnal volume variations of the human brain, which is greater in the morning^47^, whereas the choroid of the eye is thickest and its axial length shortest at night^48^. These volumetric changes are in line with findings of increased brain glymphatic function^49^ and ocular CSF clearance^1^ during the night, which is mediated by aquaporin-4 water channels. It is also possible that the nocturnal increase in retinal thickness may be related to increased convection of glymphatic solutes due to increased water content.

While the brain pulsates strongly in VLF and respiratory frequency bands^8,9,50^, there is limited information on corresponding eye pulsations. However, the eye is known to have a spontaneous intraocular pressure (IOP) and venous pulsations throughout the bulbus, which track the cardiac rhythm^51^. Furthermore, eye pulsations cease when the CSF pulse pressure equals the IOP^52^. We detected this cardiac pulsation in the ON with fMREye scanning, but surprisingly its power was relatively insignificant compared to that of the other two pulsations (Figs. 1, 3, 4, 5), despite the fact that in the whole head (brain tissue, CSF, and skin vessels) data the cardiac power is relatively well detectable. The cardiac pulse was also exceptionally small both in the frontal eye camera (FEC) and MR-compatible video ophthalmoscope (MRcVO) signals, in contrast to the respiratory pulsation power. Based on our latest findings about fMREye method, one possible reason for the indiscernibility of the cardiac (CARD) band could be the still insufficient spatiotemporal resolution of our imaging techniques. Additionally, the fastest CARD pulsations may be vulnerable to interference from saccadic and other eye movements, and the 1 Hz helium pumping signal artifact may also introduce interference in some cases at this specific frequency if heart rate is around 60 bpm. More reasonable explanations why the cardiac is not clearly depicted in the eye (even though very clearly in other structures) is the presence of other noise sources covering it like eye movements, ferromagnetic iron make-up remnants in the eyelids despite make-up removal, and such.

The human ON can range in diameter from 0.96 to 2.91 mm, with a typical size of 1.5 mm in adults^48^. Also, the ON size decreases towards the optic canal from the origin at the globe^53^. To minimize partial volume effects, we require a correspondingly small slice thickness and voxel size to minimize partial volume effects for more accurate measure of ON pulsations in fMREye. A two-slice scan yielded a stronger signal and higher FFT power for all three physiological pulsations compared to a one-slice scan. Also, the flickers were of significantly lower amplitude with simultaneously recorded 2-slice FEC and MRcVO signals, due to the smaller optic camera interference effect arising during two-slice scanning.

After settling upon two-slice scanning, we then proceeded to compare results with 3-, 4-, and 5-mm slice thicknesses. We obtained stronger whole brain means of FFT power amplitude for VLF and CARD pulsation bands in two slices of 3 mm thickness. It is noteworthy that the RESP and CARD pulsations of the fMREye data both matched the physiological measurements of respiratory belt and cardiovascular SpO2-pulseoximeter, with nearly perfect agreement (R = 0.99 and p < 0.0001). A ROI analysis of the ON indicated stronger FFT power of the VLF and CARD bands for 2-slices of 3 mm thickness. However, respiratory power in the ON was not affected by the slice thickness in any of our measurements. In future the time domain correlations of cardiorespiratory eye signals need to be verified by physiological monitoring (MR Resp Belt & Card SpO2 monitoring) with in larger numbers of subjects with precise signal triggering that we did not have in this study. However, already here the correlation of the peak FFT frequencies is 0.99 strongly indicating an accurate match with the physiological signals, which is matched by the good time domain correlation of an example case, c.f. Fig. 5G,H.

The orientation of the ocular axis, i.e., the gaze direction, within the scanner is especially important during the fMREye recordings since the orientation of the ON will follow the ocular axis^54^. A neutral position of the eye axis would allow the slice positioning to have the widest coverage of the ON, importantly covering its entire diameter including the sheath. When the participants are looking up or down, the coverage of the ON declines, such that pulsation results may change due to transient stretching of the ON and ocular structures^44^.

Eye tracking by EEG^30^ or an MR-compatible video camera^31^ are commonly employed to monitor pupil diameter, gaze direction, or vigilance during MRI^32^. MR-compatible eye tracking cameras record gaze position during scanning with high temporal and spatial resolution, and hence enable the investigation of gaze-related brain activity^55^, often due simply to their unavailability in research or clinical settings. Indeed, MR-compatible cameras are expensive, require trained staff, and costly setup and calibration time, while imposing experimental constraints, such as the need for eyes open^33^. However, we consider this technology to augment importantly the scope of fMRI investigations.

In our study, we utilized FEC to detect physiological eye pulsations during the fMRI brain acquisition, following upon pioneering studies of Li et al. employing a frontal eye tracker to record pulsatility of the eye surface^56^ and likewise the pupil edge. We found that FEC captured the respiratory pulse on the orbital surface, but the cardiac pulse was elusive due to several reasons. First, the presence of noise pulses such as eye lid closure during blinks and eye saccade movements made its detection challenging. Second, in some cases there was also some destructive interference from respiratory pulse harmonics at multiples of the principal frequency of respiration. Although we applied a low-pass filter to remove low-frequency components, there was no detectable heartbeat within the eye, and only the CARD pulse band inside the brain was visible. We were able to filter out undesirable reflections from the eye's surface, eyelid, and face skin using the *fslroi* function. In three subjects, FEC and fMREye simultaneously detected the same strong respiratory range pulsations in the human eye.

Previously, retinal video sequences have been proposed to be useful for the observation of spontaneous venous pulsation (SVP), a phenomenon arising from changes in the pressure gradient between intraocular and intracranial pressure during the cardiac cycle. SVP is usually observable within the ON head in healthy eyes^57^. In this study, we analyzed the capability of simultaneous fMREye and MRcVO for detecting physiological eye pulsation mechanisms. Based on classical understanding of neuro-ophthalmic pulsations, we predicted that cardiac pulsation should be the dominant feature in the eye. However, we were unable so far to confirm this hypothesis with MRcVO, possibly due to masking of the pulsation by eye movements and insufficient spatial resolution, given that such pulsations are indeed observable more sensitive, eye-dedicated video ophthalmoscopy outside the MR room Faraday cage^58^. More reasons would be the noise in the recordings, irregularities in light distribution of the retina due to eye lid movements, reflective artifacts covering part of optic nerve head area, etc. The ophthalmological analog to digital setup needs to be optimized well to provide reproducible sensitivity to cardiac-related pulsations. Although, the technology used can be more accurate and sensitive by improving signal and contrast to noise ratio if we do retinal imaging outside the MR room Faraday cage.

The clearest evidence for eye saccade problems was in that motion artifacts attenuated the recorded eye pulsatility by MRcVO. We applied FSL *Mcflirt* to all imaging data (fMREye, FEC, and MRcVO) after transformation into nifti format, but this approach was unfit to correct the saccade motion in most (9/12) FEC/MRcVO recordings. This may be due to the optimization of *Mcflirt* for 3D BOLD image registration rather than the present 2D imaging. However, we used AFNI *3dDespike* for removing the effects of eyelid closure during blinking, And, finally we used a more eye-movement targeted GitHub (Retinal Imaging Toolbox, https://github.com/umn-milab/retinaimagingtoolbox) retinal imaging registration code for improved correction of the motion of the retinal images^18,19,59^. The video recording occasionally exhibits an artifact in the central region of the ROI (region of interest). To mitigate the impact of this artifact, we implemented specific measures. Employing the *3dPeriodogram* function, we generated a 3dPeriodogram map, which effectively distinguished the frequency associated with the hot spot artifact. Subsequently, utilizing the *fslroi* function, we filtered out this specific frequency, effectively eliminating the artifact's influence on the subsequent FFT power analysis.

Eye movements introduced large artifacts to eye dynamic scanning, thus substantially hindering our pulsatility data analysis. The fMRI of the human retina is disfavored due to eye motion and limited spatial resolution due to weaker magnetic field gradients^60^. Plöchl and colleagues reviewed the properties of eye movement artifacts, including cornea-retinal dipole changes, saccadic spike potentials, and eyelid artifacts, and reported on their interrelations^61^. In concordance with earlier studies, they found that these artifacts arose from independent sources, with effects on the measured signal that depended on electrode site, gaze direction, and choice of reference sources.

By optimizing the fundus system and utilizing the data obtained from existing commercial fundoscopy equipment outside the MR bore, we can enhance the applicability and ease of scanning while achieving improved resolution and contrast data. This, in turn, enables better data analysis. However, it is important to emphasize that we are presenting our initial experiences with new methods, and that we encountered particular challenges when working with older volunteers, namely that lower response to the eye dilation drop as we experienced. Additionally, simultaneous fMRI scanning proved to be highly challenging, but improved design and cameras should eventually overcome this limitation. In particular, we aim to reduce eye motion artifacts by stabilizing the ophthalmic imaging system in real-time^62^. Irrespective of various adjunct devices, using a filtering algorithm to enhance the signal-to-noise ratio increased the FFT power of the physiological pulsation bands of interest and removed eye movement artifacts^63^, which presents another focus for future work^64^.

By determining the correspondence of respiratory pulses in all recording devices, it should prove possible to perform FEC and MRcVO examinations outside the MR room with a general video recording to investigate eye pulsation. Also, comparing glaucoma and idiopathic intracranial hypertension (IIH) patients with control groups presents an attractive research topic. To enable eye closed scanning and motion correction algorithms within MRcVO calls for improved motion control for the MRcVO recordings. The aquaporin-4 channels that drive glymphatic flow are affected by specific antibodies in neuromyelitis optical-spectrum diseases including bilateral optic neuritis and spinal cord myelitis^45,46^, presenting yet another target for the present methods.

There are five diverse types of eye movements contributing to motion artefacts: saccades, smooth pursuit, and vestibular, optokinetic, and vergence eye movements^65^, which can produce rotations of 40° in only 100 ms^65^. How these movements affect the dynamics of ocular fluids is only vaguely understood, although micro saccadic oscillations are related to dynamic changes in the vitreous humor^66^. Eyelid closure^37^ and saccadic movements also modify also downstream brain neuronal network connectivity^38^ and the amplitude of resting-state brain fluctuations^39^, thus presenting additional research targets in addition to the effects of eye lid motion on solute transport of the eye itself.

Commonly, efforts to reduce eye movement signals arise in the context of BOLD contrast scan signals, but these same movements may be drivers for solute movement. Thus, we perceive an opportunity to establish better the nature of eye physiological pulsation bands in a multimodal approach. Better motion control may help in obtaining more reliable data from the retina through a projection-artifact removal algorithm^61,63^.

## Conclusions

We developed a simultaneous multimodal brain and eye scanning system that enables the detection of especially vasomotor and respiratory neuro-ophthalmic pulsations driving glymphatic solute transport in the healthy human eye using fMREye, FEC, and MRcVO. Two-slice fMREye imaging with thickness of 3 mm provided the least artifacts and was most sensitive in detecting pulsation powers in the eye. The fMREye pulsation data accurately reflected physiological cardiorespiratory recordings from the whole head. However, despite the clear evidence of cardiac pulsations in the brain and skin, the cardiovascular band was surprisingly small in the eye structures compared to the VLF and respiratory pulsations. Despite challenges such as eye movements and limitations in spatial and temporal resolution, fMREye offers unique advantages over optical imaging methods, including its non-invasive and tracer-free nature, complementary information, compatibility with MRI, and potential clinical applications. Further technical improvements are needed to enhance the imaging of eye pulsatility, including better motion control techniques and the utilization of projection-artifact removal algorithms. The optimization of fMREye holds promise for clinical applications in the diagnosis and monitoring of ocular and neurological conditions.

The study also provides insights into the diurnal variations and neuro-ophthalmic pulsations in the eye. Our findings highlighted the importance of the ON diameter, slice thickness, and gaze direction in capturing accurate pulsation data. Additionally, the use of MR-compatible eye tracking cameras was discussed as a valuable tool for monitoring eye movements during MRI. Future research directions include investigating the effects of eye pulsations in different patient populations, exploring the relationship between eye pulsations and glymphatic solute transport, and improving motion correction algorithms for eye imaging. Overall, this study contributes to the understanding of eye physiology and opens avenues for further investigations into the pulsatility of the human eye.

## Materials and methods

The study population comprised 16 healthy volunteers (5 males and 11 females of mean (SD) age 38.7 ± 12.4 years). We completed the analysis in all sixteen subjects, with duplicate scans in two individuals. The study and the applied fMREye, MRcVO, and FEC technology were approved by the Regional Ethics Committee of the Northern Ostrobothnia Hospital District (FIMEA/2021/002535). The study was performed in Oulu University Hospital, University of Oulu, Oulu, Finland. We obtained written informed consent from all participants, according to the requirements of the Declaration of Helsinki. All procedures were performed in accordance with relevant guidelines. All subjects were healthy and met the following inclusion criteria for multimodal scanning: no continuous medication, no neurological nor cardio-respiratory diseases, non-smokers, and no pregnancy.

### Data acquisition

Subjects were imaged in Siemens MAGNETOM 3T Vida scanner (Siemens Healthineers AG, Erlangen, Germany) using 64-channel head-coil. We used the following scanning parameters for the Siemens ep2d GRE-EPI (2D echo planar imaging) BOLD sequence: repetition time (TR = 100 ms), echo time (TE = 15 ms), echo train length (ETL:32), flip angle (FA = 15°), 128*128 matrix yielding 2.64 × 2.64 mm pixels, and one or two slices of varied thickness: (3, 4, and 5 mm). For structural alignment, we obtained anatomical 3D 0.9 mm cubic voxel images from T1-MPRAGE (TR = 1900 ms, TE = 2.49 ms, FA = 9°) and T2-spc (TR = 3200 ms, TE = 412 ms, FA = 90°) with 240 mm^3^ field of view (FOV).

Twelve of the 16 subjects (5 males and 7 females of mean (SD) age 42.5 ± 14.4 years) were scanned for 3 min epoques with simultaneous eye-scanning by either MRcVO or fMREye. The MRcVO subjects’ left eye pupil was dilated with tropicamide (OFTAN TROPICAMID, 5 mg/ml) eye drops. The remaining six subjects (all males, of mean (SD) age 33.8 ± 9.1 years) were scanned with the functional eye camera (FEC) during fMREye imaging of the eye surface structures, but without pupil dilation. Two subjects underwent both MRcVO and FEC with fMREye scanning on separate days. Eye scanning commenced after the pupil had been dilated some 3–5 min after drug administration. Multimodal imaging was also used to measure the physiological pulsations, along with scanner respiratory belt and right index fingertip SpO2 pulse oximeter^67^ recordings from the group of 12 subjects.

**Figure 6:**
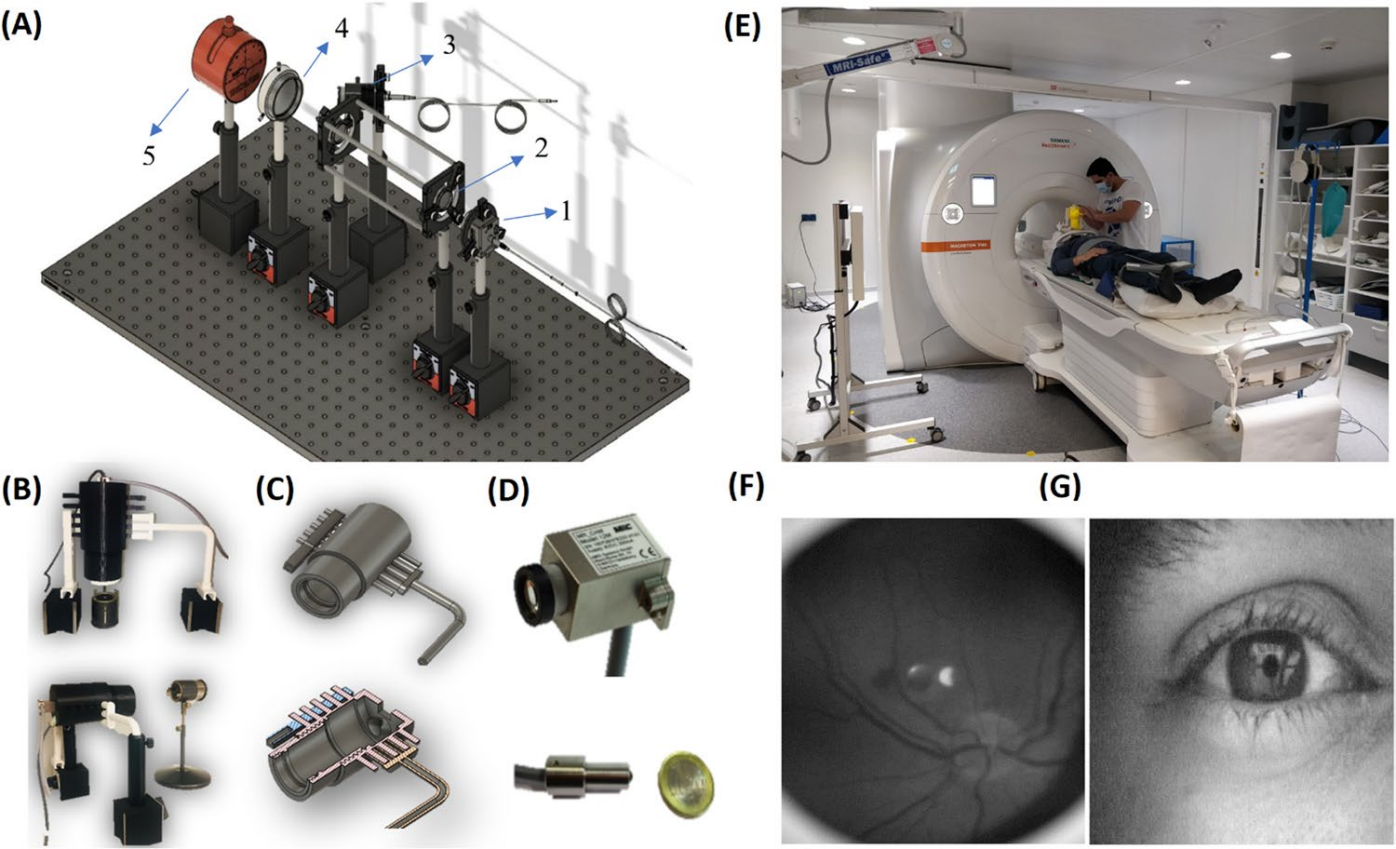
Technology and development of the eye scanning setup. (**A**) Optical equipment for the experimental testing of fundoscopy. 1. The MR compatible video camera, 2. Zoom lenses 3. MR compatible white LED (light emitting diode), 4. a 20D/28D lens, 5. Artificial eye model. (**B**) Third version of the prototype fundoscopy system for vertical and horizontal positioning of MR-compatible video ophthalmoscope (MRcVO). (**C**) ABS material 3D-printed optical system holder for use inside the MR scanner room’s Faraday cage. (**D**) MRC’s (R) MR compatible camera, and MR compatible LED (light emitting diode) light source. (**E**) Human fMREye scanning in a 3 T Vida MRI laboratory. Example of image of (**F**) MRcVO video image illustrating the human retina, c.f. Supplementary file for simultaneous fMREye/ophthalmoscopy mpeg-video, and (**G**) functional eye camera (FEC) video taken during simultaneous 3T fMREye scanning.

This system is the first MR-compatible video ophthalmoscope, which enables continuous video recording from the retina. The MRcVO is an optical system that includes, as shown in Fig. 6A: 1. The MR compatible video camera (MRC Systems GmbH, Heidelberg, Germany), 2. Zoom lenses 3. MR compatible white LED, and 4. a 20D/28D lens (Volk Optical Inc., Mentor, OH, USA), which were assembled inside an 3D printed acrylonitrile butadiene styrene (ABS) optical system holder of in-house construction (FABLAB, University of Oulu, Oulu), (Fig. 6B–D). Due to individual differences in pupillary dilatation (practically, less pupillary dilation response for older subjects), we scanned the retina with two different optical arrangements. In the first arrangement, for those with pupil diameter greater than 4 mm open, we used a 20 diopter Volk lens placed 13 cm in front of an MRC camera equipped with an 8 mm zoom lens. In this setup, the FOV was 30 degrees, with an optimal distance between the eye and the fundoscopy of about 5 cm. In the second arrangement for individuals with pupil measuring less than 4 mm, we used a 28 diopter Volk lens positioned 15 cm in front of the MRC camera equipped with a 12 mm zoom lens. For this optical setup, the FOV was 60 degrees, with the optimal distance between the eye and the fundoscopy of about 3 cm.

The light source, consisting of a white LED and a blue high-pass filter to remove wavelengths less than 500 nm, was aligned under the camera at an angle of 30 degrees, such that the reflected rays of the lens surface scattered to the surrounding side area and did not return to the camera, thus uniformly illuminating the surface of the retina with white light. In our setup, the incident light enters the eyeball from the sides of the pupil (towards the posterior part of the eye). The reflected light exits from the center of the pupil and is directed to the MRC camera by the lenses, with passage through a filter box. The filter box prevents the transmission of extraneous signals into the MR cabinet, thus minimizing interference with the video signals and MRI imaging. Signals are recorded digitally via a video card. Unlike in conventional ophthalmoscopes, we simplified our system to enable its use inside the MR bore to capture images from the retina and eye surface simultaneous fMREye (Fig. 6E–G), by placing the light source in the same path as the optical imaging system. So designed, the system occupies a smaller space, which does not interfere with installation of the Siemens 64-channel head coil. The intensity of the retina was measured across the optic disk and couple of millimetres around it included all the structures to statistical comparison and using selected pixel to show the charts with sharpest peaks.

**Figure 7:**
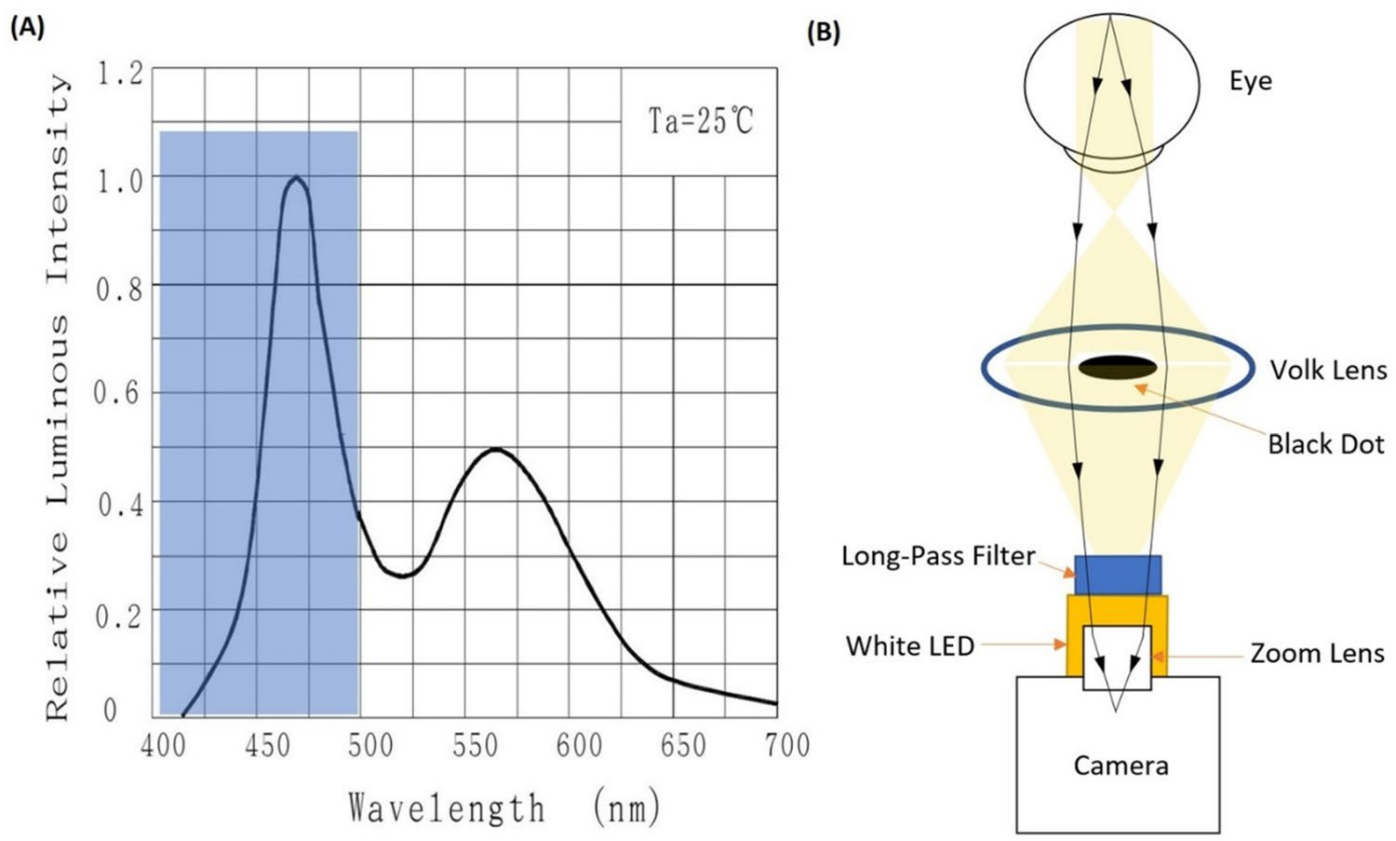
(**A**) The light source spectrum, and the filtered region of wavelength < 500 nm. (**B**) Optical design schematic and ray tracing.

We tested the system using an artificial eye model^68^ as illustrated in Fig. 6A. A 3D model can be found on our webpage (https://www.oulu.fi/en/research-groups/oulu-functional-neuroimaging-ofni). The continuous measurements call for low light intensity during the prolonged illumination, especially given the pharmacological pupil dilation. The MRcVO system contains a source for visible light (400 to 700 nm), as shown in Fig. 7. The spectrum of the MR-camera light source raises the possibility of blue-light photochemical and thermal injury of the eye. We obviated this risk through long-pass filtration of blue and ultraviolet light (wavelength < 500 nm) projected by the LED^69^ and scrupulous control of light intensity. To meet and exceed eye safety regulations, we set the light intensity to a maximum of 2700 lx, which we confirmed by measurement to be below threshold^70^. The 30-degree FOV ophthalmoscope provides a single-window from the retina, including the optic nerve head and fovea, in single-shot simultaneous MR scanning^71^. Our optical system has a manual variable focus lens and an adjustable 3D-printed ABS material holder that allows adjusting the optical system and changing the position of the lenses and distance between the device and the participant’s eye to provide optimal resolution and to direct the light inside the eye without inference in positioning of the 64 channel head coils irrespective of head size and position inside the bore.

To afford easy application of the optical device, we designed an optical setup with a single path for all light rays. Thus, the LED light projected inside the eyeball, and the light reflected from the retina follows the same path. We used a 2 mm diameter black paint dot on the surface of the Volk lens to eliminate the reflected LED light from the center of the focusing lens toward the camera, which is a problematic aspect of conventional ophthalmoscopes. The LED had the lowest light that was sustainable for long video recordings, which was directed inside the eye such that the operator could adjust the system as required during the imaging.

The maximum light intensity in the focal point area was ~ 7800 lx (The supplementary file, Table 1), corresponding to power of 1 mW/cm^2^ at 555 nm wavelength. According to a previous report, the safety limit for 400–700 nm illumination is (a) a power of 2 mW/cm^2^ for direct ophthalmoscopy with 500 s maximum cumulative exposure and (b) and energy of 10 mJ/cm^2^ for times between 10 and 1000 s^70^, which is an order of magnitude below the damage threshold for visible wavelength laser light. On the other hand, the focal area is the entire cornea surface, which presents a surface area of about 1 cm^2^, thus favoring the safety margin of our set-up. Also, the light source spectrum range of our white LED, there is less than ~ 32% energy absorption on the cornea surface, suggesting by sample calculation that illumination with 1 mW/cm^2^ deposits only 0.32 mW/cm^2^.

### ROIs and masking

For the FEC data, the recorded video had a duration of 30 s, while the MRcVO data was obtained over 3 min. To analyze the time signal and FFT power from these videos, specific Regions of Interest (ROIs) were carefully chosen. Following data registration and processing, individual pixels for FEC and MRcVO were selected, along with a voxel for fMREye, to clearly illustrate FFT power peaks and time-courses. The ROI for FEC data encompassed the region around the pupil (Fig. 5A), and a representative pixel within this region was chosen for analysis. In the case of MRcVO data, the ROI consisted of a circular area around the optic disc, with a radius ranging from 1 to 2 mm, which included the retinal surface and associated vessels. Subsequently, after image registration, a pixel within this ROI was selected for further analysis. Regarding the fMREye data, a different approach was taken. We initially performed image registration and processing. Subsequently, we employed masking to isolate specific areas of interest. For the optic nerve, the clearest portion near the eye globe was selected using anatomical T2-weighted MRI data, as depicted in Fig. 4A. For the eye surface, we selected a voxel based on the mask illustrated in Fig. 5A. Furthermore, for the retinal analysis, a Region of Interest (ROI) was defined based on Fig. 5B, focusing on the optic nerve head area.

All data were consistently acquired from the left eye for this study.

### Data pre-processing

The data were analyzed based on the ep2d fMREye signal, MRcVO, and FEC images. The fMREye data were preprocessed and analyzed using FSL (5.09 BET software)^72^, AFNI (analysis of functional neuroimages, v2)^73^, and MATLAB (R2019). The functional data preprocessing was performed using the FSL pipeline. After removal of the 20 initial frames, data were high-pass filtered with a cut-off frequency of 0.008 Hz (125 s), and framewise head motion correction was then performed with FSL 5.08 MCFLIRT software^74^. The AFNI *3dDespike* function was used to remove spikes from the data.

The eye camera software (MRC eye-tracking software) records video from the eye at a resolution of 640 × 480 pixel at rate of 30 frames per second, and outputs the resulting file in greyscale .vid format. Then, we used a custom MATLAB script to extract each video frame and transform it into AVI format, and nifti format using the *niftiwrite* function. Then, by using the GitHub algorithm (https://github.com/umn-milab/retinaimagingtoolbox)^18,19^ for motion correction, we corrected the motion of the cropped videos and then converted the AVI file to NIFTI format. To perform image registration, we employed the Git-hub image registration algorithm with the following input parameters:

- mask: [], indicating no mask applied.
- index_for_reference_frame: 1, signifying the first frame as the reference image.
- Kvessels: 1, specifying the number of vessels to track.
- ignore_borderPC: 10, excluding a border of 10 pixels from phase correlation analysis.
- ignore_borderLK: 10, excluding a border of 10 pixels from Lucas-Kanade tracking.
- NwinLK: 31, setting the window size for Lucas-Kanade tracking to 31 pixels.
- RGB_flag: 0, processing the input video as grayscale images.

The video file in .AVI format was provided as input, and the entire image was utilized as the region of interest (ROI). After registration, the Region of interest was cropped and then a mask was applied to exclude a few pixels in the hot spot at the center of the image, enabling further focused analysis in AFNI. As well as, by using *3dDespike* command eye blinking frames effect was removed and finally by removing edges of videos by *fslmaths.* Finally, the individual frames were merged back into a video using the FSL function *fslmerge* prior to further analysis using various FSL and AFNI (Analysis of Functional Neuroimages) software.

### Data analysis

All data from fMREye, MRcVO and FEC in Nifti-format were analyzed to achieve the FFT power spectra of the data using the AFNI *3dPeriodogram* function, which estimates the power distribution of each voxel time signal Global FFT spectra were then made by calculating a mean spectrum over the whole head using the *fslmeants* function, which gathers all power spectra in a specified ROI (region of interest) and outputs an average power spectrum. Then we used FSLeyes to visualize power spectra maps of eye and brain physiological pulsations. Data analysis was performed using FSLeyes, MATLAB, local multimodal software NAPP (nifty app for fMRI data processing) and GraphPad Prism 9 software.

The very low frequency (VLF) band for eye and brain was 0.01–0.1 Hz for every subject, while respiration (RESP) and cardiac (CARD) bands were 0.1 Hz wide, and centered around the individual peaks (i.e., peak ± 0.05 Hz). Source power was defined as the sum/integral over the frequency band of interest. This was calculated by extracting the frequency bins using the *fslroi* function and then summing the remaining bins with AFNI *3dTstat*. Respiratory belt data and cardiovascular SpO2 served for physiological verification of the accuracy of fMREye. To evaluate signal power in our analysis, we employed FSL. For the EPI slice data, a window length (NFFT) of 2048, which represents the next power of 2 of the signal length in samples, was utilized. In the case of videos, we employed NFFT = 3000 to align with the video frame rate of 30 frames per second. The spectral-temporal resolution, denoted as Δf, was calculated as the sampling rate divided by the NFFT, yielding a resolution (Δf_FEC_ = 0.01 Hz, Δf_MRcVO_ = 0.01 Hz, Δf_fMREye_ = 0.005 Hz). This value determines the frequency bins at which signal power was assessed.

To estimate signal power (P), we used the 3dPeriodogram algorithm from AFNI which is based on the standard signal processing formula:

$$ {\text{P}}\left( {\text{f}} \right)\, = \,\left( {{1}/{\text{N}}} \right) \, \left| {{\text{F}}\left( {\text{f}} \right)} \right|^{{2}} . $$

However, it uses a different normalization function called K which replaces the (1/N),

$$ {\text{P}}\left( {\text{f}} \right)\, = \,{\text{K}}\left| {{\text{S}}\left( {\text{f}} \right)} \right|^{{2}} $$

Where P(f) represents the power spectrum at frequency f, S(f) is the Fourier transform of the tapered data, i.e., ${\text{S}}({\text{f}})={\sum }_{{\text{n}}=0}^{{\text{N}}-1}{\text{w}}\left({\text{n}}\right)\times {\text{x}}({\text{n}}){{\text{e}}}^{-{\text{i}}2\mathrm{\pi fn}}$ where x(n) is the original data point at sample n and w(n) is the tapering weight applied to the data point x(n)x(n), which is defined using the Hamming window function. K is the normalization factor, which is the sum of the squares of the tapering weights, K = ∑w(n)^2^. This formula indicates that the periodogram is the squared magnitude of the Fourier transform of the weighted (tapered) data, normalized by the sum of the squares of the taper weights^75–77^.

### Correlation analysis

In the analysis, we computed time domain correlations within the respiratory signals with the xcorr function in MATLAB. Figure 5H displays the correlation among the MR respiratory monitoring belt, fMREye, and MRcVO signals in one subject. Due to the limited duration of FEC data, consisting of 30-s clips, and the presentation of example signals corresponding to frequency spectra in Fig. 5C, we supplemented the information with a manually shifted example from one subject. This additional illustration in Fig. 5G underscores the strong correlation among FEC, fMREye, and the respiratory belt signals on a time scale.

### FFT normalization procedure

As FFT spectrums of different modalities were not in the same scale, we normalized the spectra of each modality to range between 0 and 1, thus enabling visual evaluation. The values in a dataset were normalized using the following formula:

$$ z_{i} \, = \,\left( {x_{i} {-}\min \left( x \right)} \right)/\left( {\max \left( x \right){-}\min \left( x \right)} \right) $$

Where, z_i_ is the ith normalized value in the dataset, x_i_ the ith value in the dataset, min(x) is the minimum value in the dataset, and max(x) the maximum value in the dataset.

### Statistical analysis

The statistical significance of the slice number differences was conducted using data from the six subjects in whom we obtained 1 and 2 slice data. We used the two-tailed Student’s t-test (significance level Bonferroni-corrected for three frequencies p < 0.05) for hypothesis testing between the study groups in VLF, RESP, and CARD bands (sum FFT power over the frequency band of interest) and the Shapiro–Wilk test for normality. The slice thicknesses data analyses were performed using the Kruskal–Wallis one-way analysis of variance method (ANOVA) and post-hoc (Tukey) tests between different slice thicknesses (sum of FFT power) in VLF, RESP, and CARD bands in six subjects for the FEC synchrony fMREye data and 12 subjects for the MRcVO synchrony fMREye data. The statistical data were analyzed with MATLAB and GraphPad Prism 9 software.

Individuals in Fig. 6 understand that they may be identified from photographs. The written informed consent for publication was obtained from all participants.

### Supplementary Information

Supplementary Video 1.

Supplementary Information.

Supplementary Video 2.

Supplementary Table 1.

Supplementary Table 2.

## Data Availability

The datasets used and/or analyzed during the current study are available from the corresponding author on reasonable request.
