# Supplementary material for: Synchronous functional magnetic resonance eye imaging, video ophthalmoscopy, and eye surface imaging reveal the human brain and eye pulsation mechanisms
Source: Sci Rep. 2024 Jan 26;14:2250. doi: 10.1038/s41598-023-51069-1 (PMC10817967; doi:10.1038/s41598-023-51069-1)
Supplement: Supplementary file 4 — Supplementary Table 1. [file 41598_2023_51069_MOESM4_ESM.docx]

| **Electric current intensity (based on dimer marker)** | **Light intensity in focus point area (LUX)** |
| --- | --- |
| **Min** | 2750±10 |
| **Middle down** | 3300±10 |
| **Center** | 4650±10 |
| **Middle up** | 7800±10 |
| **Max** | 18950±10 |

Table 1 - Changes of light intensity on focus point area for electrical flow variations at 20 centimeters (8 inches) distance from the light source
