# Supplementary material for: Synchronous functional magnetic resonance eye imaging, video ophthalmoscopy, and eye surface imaging reveal the human brain and eye pulsation mechanisms
Source: Sci Rep. 2024 Jan 26;14:2250. doi: 10.1038/s41598-023-51069-1 (PMC10817967; doi:10.1038/s41598-023-51069-1)
Supplement: Supplementary file 5 — Supplementary Table 2. [file 41598_2023_51069_MOESM5_ESM.docx]

|  | **fMREye** | | **FEC** | | **MRcVO** | |
| --- | --- | --- | --- | --- | --- | --- |
|  | Frequency (Hz) | Peak power | Frequency (Hz) | Peak power | Frequency (Hz) | Peak power |
| Case 1 | 0.22 | 0.85 | 0.22 | 0.89 | --- | --- |
| Case 2 | 0.24 | 0.93 | 0.24 | 0.88 | --- | --- |
| Case 3 | 0.27 | 0.86 | 0.27 | 0.88 | --- | --- |
| Case 4 | 0.18 | 0.96 | --- | --- | 0.18 | 0.99 |
| Case 5 | 0.21 | 0.75 | --- | --- | 0.21 | 0.96 |
| Case 6 | 0.34 | 0.43 | --- | --- | 0.34 | 0.91 |

Table 2 - The fMREye results with respiration belt and oximeter recordings, a comparison of FEC and MRcVO data to fMREye imaging data showed identical peaks in the RESP band.
