# Supplementary material for: Synchronous functional magnetic resonance eye imaging, video ophthalmoscopy, and eye surface imaging reveal the human brain and eye pulsation mechanisms
Source: Sci Rep. 2024 Jan 26;14:2250. doi: 10.1038/s41598-023-51069-1 (PMC10817967; doi:10.1038/s41598-023-51069-1)
Supplement: Supplementary file 4 [file 41598_2023_51069_MOESM2_ESM.odt]

Supplementary

- Eye safety:

Eye safety was a paramount consideration in the project. LED (Light Emitting Diode) illumination power must be strictly maintained within a safter threshold. In this regard, we remove blue light below 550 nm wavelength (Fig 1.) using a long-pass filter to prevent any possibility of eye damage.

Figure 1. MRC’s MR-compatible LED light spectrum obtained from the device producer.

Adverse Effects of Intense Optical Radiation on the Eye

The ﬁve principal types of hazards to the eye from optical sources—whether the sources are lasers or incoherent, are listed below along with any special considerations relating to ophthalmic instrument use:

1. UV photochemical injury to the cornea (photokeratitis) and lens (cataract) of the eye (180 to 400 nm). In deriving the exposure limits (ELs), the underlying assumptions were (a) that exposures could be averaged over a 1-mm aperture at the corneal plane for pulsed exposures lasting less than approximately 0.3 s, whereas, for CW (Continuous Wave) exposures lasting more than 10 s, the averaging aperture would increase to 3.5 mm; and (b) that there should be no unusual focusing of radiant energy within the lens (as could occur during Maxwellian view). Thermal damage to the cornea and lens is rarely of concern in the UV region, except from a laser beam of high irradiance. However, the underlying assumption that relative eye movements during normal vision will exceed a 1-mm area is not valid for ophthalmic examinations. Hence the 1-mm aperture should be applied for CW exposures to radiant energy from ophthalmic instruments during the use of which the head or eye position may be restrained.

2. Blue-light photochemical injury to the retina of the eye (principally 400 to 550 nm; unless aphakic, 310 to 550 nm). Retinal injuries have been reported to result from retinal exposures to the light from operating microscopes during cataract surgery. The underlying assumptions in deriving the ELs for the normal eye were (a) a 3-mm pupil diameter resulting from staring at a bright light and (b) an intact crystalline lens. However, during cataract surgery, after the lens has been removed and before insertion of an artiﬁcial intraocular lens (IOL), the normal ﬁltration of UV radiation (UVR) by the lens is not present, thus potentially exposing the retina to near-UVR (310 –400 nm) from the light source. If the pupil is dilated by mydriatics, the retinal hazard will be increased. The retinal averaging area should be 30 µm for a stabilized image, which could occur during ocular surgery, or 180 µm for normal, awake (unstabilized -image-) viewing conditions, which corresponds to the 11-mrad angular averaging ﬁeld of view for measuring blue-light effective radiance.

3. Retinal thermal injury of the eye (400 to 1400 nm). The underlying assumptions in deriving the ELs were (a) that a 7-mm aperture for averaging corneal irradiance and a smaller 3-mm pupil existing for exposure durations greater than ~1 s was necessary and (b) that eye movements would compensate for any potential increased risk for exposure durations longer than 5–10 s. However, the pupil may be 8 mm during the use of mydriatics, and eye movements may be reduced or stabilized during an examination. The related near-IR thermal limit (770-1400 nm) was based on the underlying assumption that direct viewing could take place for periods of 10 s or longer without an aversion response (reduced visual stimulus) and that a 7-mm diameter pupil was appropriate. This near-IR limit corresponds to a retinal irradiance of approximately 0.7 W/cm2, which we consider to be acceptable for large retinal-area irradiation for lengthy periods. In terms of retinal irradiance distribution, the image-size dependence of retinal hazard requires analysis of image-area “hot-spots” as small as 30 µm in diameter.

4. Near-IR thermal hazards to the lens (800 to 3000 nm). The underlying assumptions were (a) that the energy is absorbed in the cornea, aqueous, and lens, and (b) that a 3.5-mm aperture was adequate for irradiance averaging for periods of 10 s or longer because of heat conduction and eye movement. No considerations were made regarding a beam waist within the lens, as would occur with Maxwellian-view illumination. For that reason, thermal model calculations were necessary to develop guidance for limiting apertures in the plane of the lens. This approach is also necessary to determine the thermal risks to the cornea and lens from pulsed, high-irradiance UV-A sources (lasers). A 1-mm averaging aperture is thus appropriate for ophthalmic instruments.

5. Thermal injury (burns) of the cornea of the eye (1400 nm to 1 mm). The underlying assumptions were (a) that the energy was absorbed in the cornea, with only slight absorption in the aqueous humor, although thermal diffusion will heat the aqueous humor, and (b) that a 3.5-mm aperture was adequate for irradiance averaging for periods of 10 s or greater because of heat conduction and eye movements. However, for a stabilized eye, a 1-mm limiting aperture is appropriate.

Note: In MRcVO (Magnetic Resonance Compatible Video Ophthalmoscopy) there is a light source with visible light (400 to 700 nm), as shown in Figure 8. So, based on the light source spectrum, blue-light photochemical injury to the retina and retinal thermal injury of the eye is in the realm of possibility, and therefore calls for special attention. We addressed this issue as follows: (a) by using a long-pass filter to eliminate blue light and (b) by controlling light intensity under the threshold of eye hazard, thereby eliminating the risk of retinal thermal injury.

Table 1 - Changes of light intensity on focus point area for electrical flow variations at 20 centimeters (8 inches) distance from the light source

| Electric current intensity (based on dimer marker) | Light intensity in focus point area (LUX) |
| --- | --- |
| Min | 2750±10 |
| Middle down | 3300±10 |
| Center | 4650±10 |
| Middle up | 7800±10 |
| Max | 18950±10 |

As shown in table 1, at the middle up a range of electric current intensity, light intensity in the focus point area is ~7800 lux, corresponding to 1 mW/cm2 at 555 nm. According to some reports, the safety range for 400 to 700 nm is (a) 2 mW/cm2 for direct ophthalmoscopy with 500 s maximum cumulative exposure time and (b) 10 mJ/cm2 for 10<t<1000 s is the energy or power that can be safely viewed with the eye without damage, this being an order of magnitude below the damage threshold for laser safety. On the other hand, the focus area in MRcVO is the cornea surface, and there is a wide area under illumination on the retina, such that the power of intensity is considerably lower than this amount. Also, in the light source spectrum range of the used white LED, there is lower than ~32% energy absorption on the cornea surface (20). Therefore, by simple calculation the maximum light intensity is 32% * 1 mW/cm2, equal to 0.32 mW/cm2.

 

- MRcVO (MR compatible Video Ophtalmoscope) video simultaneously fMREye scanning.

We have attached a video file (filename: Human_Retinal_Live_Video) from a live MRcVO recording from human retina.

- Human MRcVO, fMREye and FEC pulsations data

After comparing the fMREye results with respiration belt and oximeter recordings, the FEC and MRcVO data were also compared to the fMREye imaging data, revealing consistent peaks in the RESP band. Simultaneous fMREye and MRcVO examinations further detected robust pulsations within the respiratory range in the human eye. This pulsatility was observed both in the eye surface and the retinal vasculature, with matching peak frequencies in the fMREye and FEC/MRcVO recording.

Table 2 - The fMREye results with respiration belt and oximeter recordings, a comparison of FEC and MRcVO data to fMREye imaging data showed identical peaks in the RESP band.

|  | fMREye | FEC | MRcVO |
| --- | --- | --- | --- |
| Frequency (Hz) | Peak power | Frequency (Hz) | Peak power | Frequency (Hz) | Peak power |
| Case 1 | 0.22 | 0.85 | 0.22 | 0.89 | --- | --- |
| Case 2 | 0.24 | 0.93 | 0.24 | 0.88 | --- | --- |
| Case 3 | 0.27 | 0.86 | 0.27 | 0.88 | --- | --- |
| Case 4 | 0.18 | 0.96 | --- | --- | 0.18 | 0.99 |
| Case 5 | 0.21 | 0.75 | --- | --- | 0.21 | 0.96 |
| Case 6 | 0.34 | 0.43 | --- | --- | 0.34 | 0.91 |

As shown in table 2 the peak frequencies in fMREye and FEC/MRcVO recordings matched each other.
